# Quantitative assessment of magnetic resonance derived myocardial perfusion measurements using advanced techniques: microsphere validation in an explanted pig heart system

**DOI:** 10.1186/s12968-014-0082-0

**Published:** 2014-10-14

**Authors:** Andreas Schuster, Niloufar Zarinabad, Masaki Ishida, Matthew Sinclair, Jeroen PHM van den Wijngaard, Geraint Morton, Gilion LTF Hautvast, Boris Bigalke, Pepijn van Horssen, Nicolas Smith, Jos AE Spaan, Maria Siebes, Amedeo Chiribiri, Eike Nagel

**Affiliations:** Division of Imaging Sciences and Biomedical Engineering; King’s College London British Heart Foundation (BHF) Centre of Excellence; National Institute of Health Research (NIHR) Biomedical Research Centre at Guy’s and St. Thomas’ NHS Foundation Trust; Wellcome Trust and Engineering and Physical Sciences Research Council (EPSRC) Medical Engineering Centre, The Rayne Institute, St. Thomas´ Hospital, London, UK; Department of Cardiology and Pneumology and German Centre for Cardiovascular Research (DZHK, Partner Site Göttingen), Georg-August-University, Göttingen, Germany; Department of Biomedical Engineering & Physics, Academic Medical Centre, Amsterdam, The Netherlands; Philips Healthcare, Imaging Systems – MR, Best, The Netherlands; Medizinische Klinik III, Kardiologie und Kreislauferkrankungen, Eberhard-Karls-Universität Tübingen, Tübingen, Germany

**Keywords:** Cardiovascular magnetic resonance, Myocardial perfusion imaging, Quantification, Deconvolution, Microspheres, Isolated heart perfusion

## Abstract

**Background:**

Cardiovascular Magnetic Resonance (CMR) myocardial perfusion imaging has the potential to evolve into a method allowing full quantification of myocardial blood flow (MBF) in clinical routine. Multiple quantification pathways have been proposed. However at present it remains unclear which algorithm is the most accurate. An isolated perfused, magnetic resonance (MR) compatible pig heart model allows very accurate titration of MBF and in combination with high-resolution assessment of fluorescently-labeled microspheres represents a near optimal platform for validation. We sought to investigate which algorithm is most suited to quantify myocardial perfusion by CMR at 1.5 and 3 Tesla using state of the art CMR perfusion techniques and quantification algorithms.

**Methods:**

First-pass perfusion CMR was performed in an MR compatible blood perfused pig heart model. We acquired perfusion images at physiological flow (“rest”), reduced flow (“ischaemia”) and during adenosine-induced hyperaemia (“hyperaemia”) as well as during coronary occlusion. Perfusion CMR was performed at 1.5 Tesla (n = 4 animals) and at 3 Tesla (n = 4 animals). Fluorescently-labeled microspheres and externally controlled coronary blood flow served as reference standards for comparison of different quantification strategies, namely Fermi function deconvolution (Fermi), autoregressive moving average modelling (ARMA), exponential basis deconvolution (Exponential) and B-spline basis deconvolution (B-spline).

**Results:**

All CMR derived MBF estimates significantly correlated with microsphere results. The best correlation was achieved with Fermi function deconvolution both at 1.5 Tesla (r = 0.93, p < 0.001) and at 3 Tesla (r = 0.9, p < 0.001). Fermi correlated significantly better with the microspheres than all other methods at 3 Tesla (p < 0.002). B-spline performed worse than Fermi and Exponential at 1.5 Tesla and showed the weakest correlation to microspheres (r = 0.74, p < 0.001). All other comparisons were not significant. At 3 Tesla exponential deconvolution performed worst (r = 0.49, p < 0.001).

**Conclusions:**

CMR derived quantitative blood flow estimates correlate with true myocardial blood flow in a controlled animal model. Amongst the different techniques, Fermi function deconvolution was the most accurate technique at both field strengths. Perfusion CMR based on Fermi function deconvolution may therefore emerge as a useful clinical tool providing accurate quantitative blood flow assessment.

## Background

Coronary artery disease (CAD) related myocardial infarction (MI) and heart failure constitute a leading cause of death in the western world [1]. Myocardial ischaemia represents the major pathophysiological substrate underlying these cardiovascular diseases and is known to be the most important predictor of negative outcome after revascularization in CAD [2]. Myocardial perfusion imaging can accurately determine myocardial ischaemia and therefore detect the physiological significance of a coronary artery narrowing which is not possible with luminal angiography [3,4]. Myocardial perfusion imaging with cardiovascular magnetic resonance (CMR) compares favourably to other non-invasive techniques [5–9] and is increasingly used for patient management and clinical decision-making [10–12]. Perfusion CMR has been validated against invasive hemodynamic measurements such as coronary flow reserve (CFR) and fractional flow reserve (FFR) [7,13–19]. The feasibility of full quantification of myocardial blood flow (MBF) with perfusion CMR has been demonstrated [20–23] and has been compared to positron emission computed tomography (PET) based quantification [24]. Even though the diagnostic accuracy for detection of significant CAD is similar between quantitative PET and CMR the perfusion estimates based on different mathematical models vary between modalities [24]. Therefore, before perfusion CMR quantification can become a clinically useful diagnostic test for non-invasive assessment of MBF [21], a number of important considerations still have to be addressed: 1) full quantification of myocardial perfusion is estimated from signal intensity (SI) time curves and the relationship between arterial input function (AIF) and myocardial response curves. Both SI time curves depend on heart rate, cardiac output, ejection fraction, and coronary anatomy and may not necessarily be the same for a given sequence or field strength. 2) There are several mathematical algorithms available for perfusion quantification based on CMR derived SI-time curves such as Fermi function deconvolution (Fermi), autoregressive moving average modelling (ARMA), deconvolution using an exponential basis (Exponential) and deconvolution using a B-spline basis (B-spline). At present it is unknown which mathematical algorithmis most accurate.

We have recently introduced a novel MR compatible explanted pig heart model that allows accurate control of regional blood flow and therefore represents an ideal vehicle for quantitative perfusion validation [25,26]. It is also free of many of the influences mentioned above which make quantification strategies difficult in living animals or patients. Furthermore, CMR results of the explanted pig heart model, which has precisely controlled blood flow rates can be verified with the state of the art imaging cryomicrotome technique [27]. This technique provides an unprecedented reference-standard for local tissue perfusion [28], via administration of differently coloured fluorescently-labelled microspheres into the bloodstream, which are quantified ex vivo from high-resolution 3D reconstructions of the organ [29].

The aim of the current study was to investigate which perfusion algorithm is most suited for quantitative analysis of perfusion CMR using this unique imaging set-up.

## Methods

### Experimental design of the study

**Figure 1:**
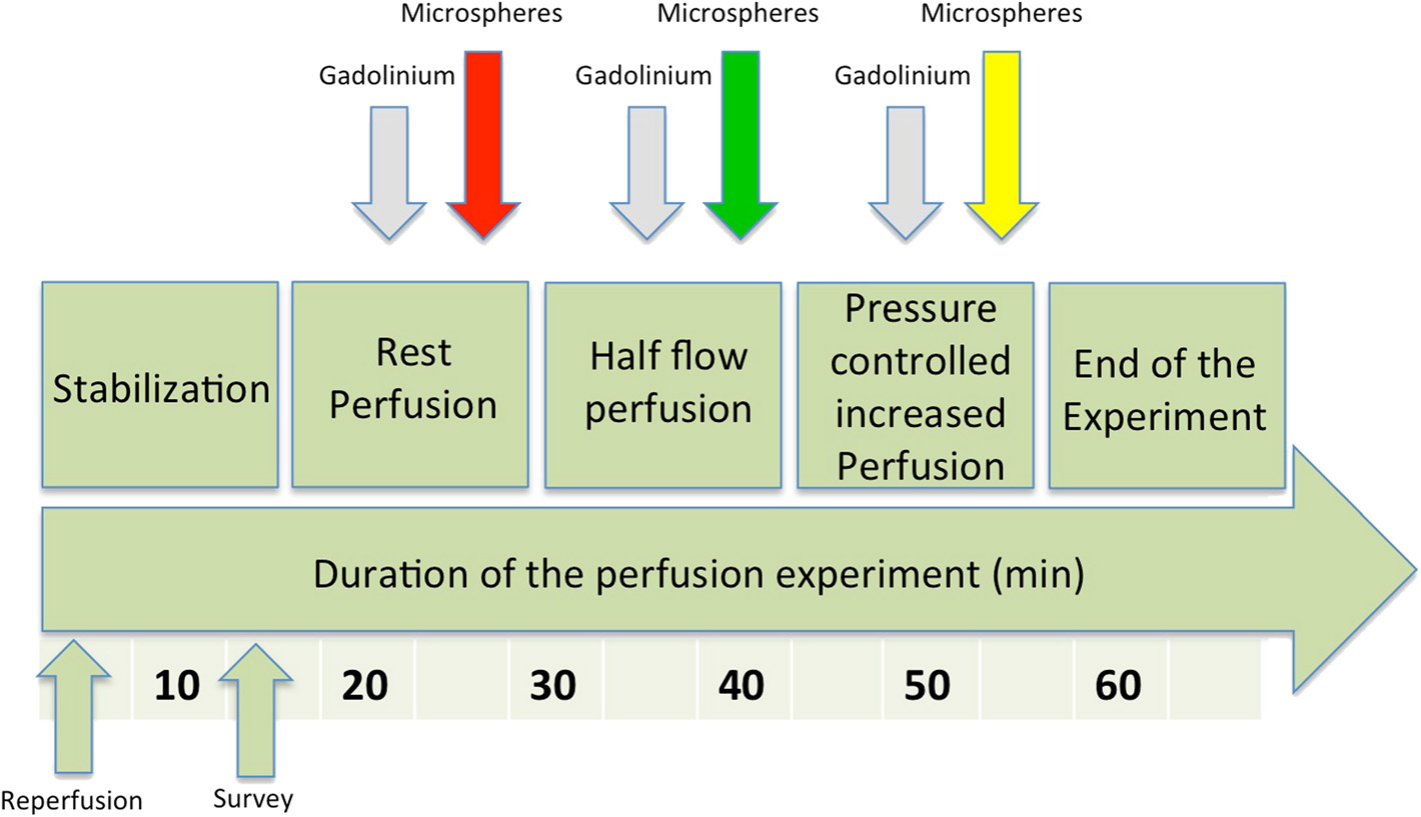
**Time-course of the CMR examination.** After preparation stability was achieved, CMR imaging was started with the acquisition of rest perfusion images. This process was repeated with a 50% flow reduction and during pharmacological vasodilation with adenosine with coronary perfusion pressure controlled increase in MBF. Microspheres were injected after each gadolinium injection with a different colour for each perfusion scan. Please note that the order of the changes made to the flow and the respective colour of injected microspheres were randomized.

All animal experiments were conducted after approval by the U.K. Home Office in accordance with the U.K. Animals (Scientific Procedures) Act of 1986 and in compliance with the World Medical Association Declaration of Helsinki regarding ethical conduct of research involving animals. Eight healthy Large White Cross Landrace pigs weighing between 41 and 54 kg were included in this study (Harlan Laboratories, UK). Hearts were harvested as previously described [25,26]. Sedation was performed with ketamine (10 mg/kg i.m.) and xylazine (0.3 mg/kg i.m.) in combination with alphaxolone for general intravenous anaesthesia (1.5 mg/kg i.v.). Heparin was administered (5,000 IU) and exsanguination started through the superior vena cava. A schematic drawing of the set-up is provided elsewhere [25]. The hearts were removed after transection of the great heart vessels and intra-coronary infusion of cold (4°C) cardioplegic solution (Martindale Pharmaceuticals, Romford, Essex, UK) was performed. Back in the MR-suite, catheters were inserted into the coronary arteries for reperfusion. The perfusion circuit was then connected to the catheters to allow continuous blood perfusion of the coronary arteries. A 3-way stopcock was used at the arterial side of the circuit for the injection of contrast agents, microspheres and adenosine. The stopcock was placed far away from the heart to allow sufficient mixing of injected substances with the perfusing blood. Special attention was drawn to precisely tape the arterial inflow tubing to the outer wall of the heart chamber to allow a perpendicular cut through the tubing within each perfusion slice acquisition to derive the AIF [25]. To create left ventricular (LV) preload a pressure balloon was inserted through the aortic valve into the left ventricle and inflated to a systolic pressure of 50 mmHg. After the hearts were cannulated, pressure controlled perfusion of the coronary arteries was started at around 50 mmHg. Arterial perfusion pressure was measured using manometers connected to the arterial inflow tubing. Over approximately 5 minutes pressure was slowly increased to a constant perfusion mode of 0.8 mL/min/g. In the event of ventricular fibrillation, electrical defibrillation was performed. After preparation stability was achieved the left anterior descending (LAD) coronary artery was occluded. The specific set-up of the explanted pig heart model and the fact that there are virtually no collaterals present in Large White Cross Landrace pigs allowed creation of a territory with a perfusion defect and a normally perfused remote territory and thus a variety of different flow values for perfusion validation within each animal. CMR imaging was started and perfusion-CMR was performed (in randomized order) at rest, with 50% flow reduction and during pharmacological vasodilation with adenosine (Figure 1). During adenosine infusion, the perfusion pressure dropped due to the induced vasodilation. To achieve hyperaemia the flow was altered to restore the same coronary perfusion pressure as during the resting state.

### Cardiovascular magnetic resonance

The animal studies were performed on 1.5 Tesla (Achieva CV, Philips, Best, The Netherlands), (n = 4) and 3 Tesla (Achieva TX, Philips, Best, The Netherlands), (n = 4) clinical MR scanners. For signal reception, a clinical interventional L-flex receiver coil array was tightly positioned around the heart chamber, which was then placed in the isocenter of the magnet. Perfusion CMR data were acquired in short axis orientation of the LV following a recognized standard model [30].

Image parameters at 1.5 Tesla were as follows: CMR-perfusion imaging was performed with a 5 fold k-t broad linear speed up technique (BLAST) accelerated balanced turbo gradient echo pulse sequence with 11 training profiles yielding a typical spatial resolution of 1.9×2×10 mm. TE/TR was 1.35/2.71; 50° flip angle; 90° prepulse and 100 ms prepulse delay.

At 3 Tesla we used a saturation recovery gradient echo pulse sequence accelerated with k–t BLAST (k-t factor 5 and 11 training profiles) with a repetition time of 2.7 ms, echo time of 0.9 ms, flip angle 20°, spatial resolution at 1.3 × 1.3 × 8 mm.

Perfusion-CMR was performed using a dual bolus scheme with 5 mL of dilute (0.007 mmol/mL) and 5 ml of neat (0.07 mmol/mL) gadobutrol bolus injections (Gadovist, Bayer Healthcare, Leverkusen, Germany) [31]. Perfusion images were acquired in three myocardial slices according to the 16 segment model of the American Heart Association (AHA) [30]. Post-processing of the perfusion images included the manual delineation of endocardial and epicardial contours as well as selecting a region of interest within the arterial inflow tubing to determine the AIF and was performed with a dedicated software prototype (Philips Healthcare, Best, The Netherlands) [25,32].

### Quantitative analysis of perfusion CMR

The LV was divided into standard segments [30]. The SI-time curves of the perfusion CMR images were incorporated into MATLAB (Natick, MA, USA) and different algorithms for quantification were applied.

These quantification methods which are based on the central volume principle [33] deconvolve the tissue signal during the first pass of a bolus of contrast agent with the arterial input function normally sampled from the LV or the arterial inflow tubing under the experimental conditions of the current study.

According to the central volume principle the concentration of the contrast agent in the tissue region *C*_*tiss*_(*t*) is related to the concentration of the contrast agent in the arterial input region *C*_*aif*_(*t*) via the following convolution equation:

$$ {C}_{tiss}(t)={C}_{aif}(t) \otimes h(t) $$

where *h*(*t*) is the unknown tissue impulse response.

The above equation needs to be deconvolved to estimate *h(t)* and therefore estimate the myocardial blood flow (MBF) according to *h*(*t* = 0).

Deconvolution methods used in this work are Fermi function deconvolution, (ARMA), Exponential basis deconvolution and B-spline basis deconvolution [21,34].

These deconvolution algorithms consist of modelling parameters and variables. The degree of splines, the number of splines and the position of the break points (control points) are the varying parameters in the B-spline function. Similarly to B-spline, the total number of exponential functions and the decay rates of the exponential functions (or in other words the placement of time scales and their distribution) are the modelling parameters for exponential basis deconvolution. With ARMA, no mathematical model is used for the representation of the tissue impulse response. The auto-regressive and moving average order (Q and L respectively) are the only factors, which have influence on the model’s accuracy. The varying parameter in Fermi is a predefined parameter, which is equal to the delay time between the arrival of contrast agent at the site of the AIF, usually the LV (or the inflow tubing in the current study) and the arrival in the myocardium. This parameter which is called tOnset depends on the placement of the individual region of interest for measuring the AIF and the tissue response [35].

It is important to note that tOnset can affect the outcome of all four deconvolution algorithms. Therefore this parameter has to be taken into special consideration when any of these methods is being used. Consequently in this study, only the intensity values after arrival of the contrast agent in the inflow tubing and myocardial tissue have been used for quantification. Effects of variation of these parameters on the outcome of deconvolution have been previously demonstrated by our group [36].

The different algorithms and their sensitivity to changes in the underlying modelling parameters are highly affected by the quality of the data. Any perturbation can affect the accuracy of the results and introduce possible variation in the output of the process. In order to render the deconvolution process more stable, reduce the computational burden and obtain the most accurate results we used 10 time scales for exponential basis deconvolution, fourth-degree B-spline polynomial with 15 equally spaced break points [36,37] and ARMA (Q = 1,L = 2) for the representation of impulse response [36]. Prior to deconvolution analysis, baseline correction, spatial and temporal filtering and homogeneity correction was performed on the extracted SI curves.

### Quantitative microsphere analysis using an imaging cryomicrotome

**Figure 2:**
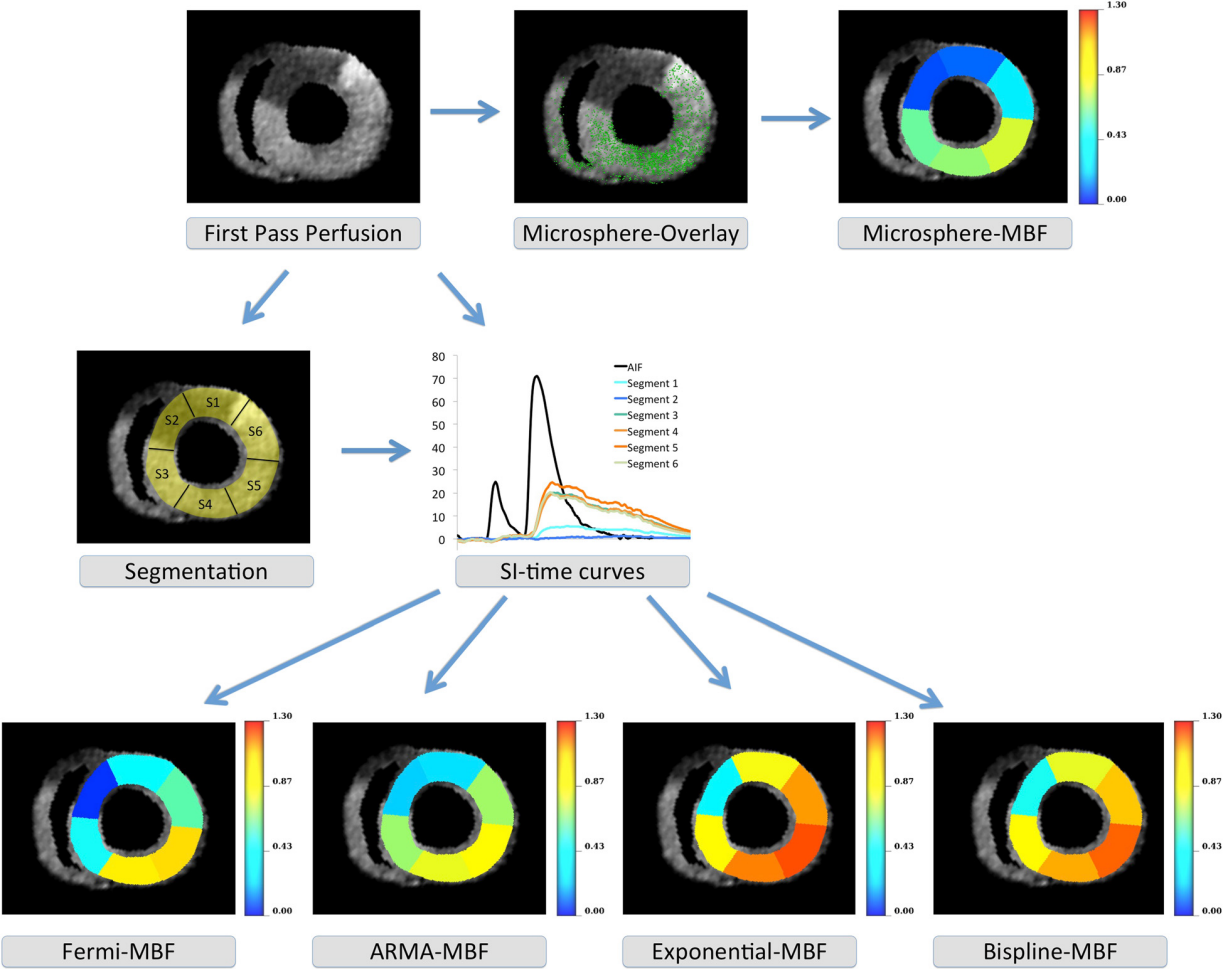
**Example illustrating the comparison of segmental perfusion by CMR and microsphere techniques at rest.** A single time frame of a perfusion study at 3 Tesla after occlusion of the LAD is shown at the upper left. The middle image (top row) shows the overlay with the microsphere distribution after transfer of the cryomicrotome images in the same coordinate space. Please note that this image provides a 2D representation of the overlaid 3D perfusion and microsphere volumes of interest as defined by the slice thickness of the perfusion CMR slices. There was good agreement between CMR and microsphere derived perfusion values without any clustering or clumping of the microspheres. Segmental microsphere quantification reveals the perfusion defect and the normal perfusion in remote myocardium (upper right corner). In the middle row segmentation of the perfusion image is displayed (left), as well as segmental signal intensity (SI) time curves during first pass of gadolinium (middle). Using the SI-time curves with different quantification algorithms delivers CMR based quantitative results [mL/min/g] depending on the algorithm used (lower row). In this example Fermi function deconvolution shows the closest agreement with the microspheres based results (lower left corner).

Microspheres used were 15 μm in size and fluorescently labelled with either of the following colours: green, yellow, red, carmine or scarlet (FluoSpheres®, Molecular Probes Inc, Eugene, Oregon, USA). Before injection microspheres were sonicated and approximately 100,000 microspheres were diluted with whole blood to a total volume of 2 ml. Immediately after the gadolinium injection microspheres were injected into the circulation over a period of two minutes (1 ml/min injection speed) at the same site used for gadolinium injection. Up to 3 different colours of microspheres were used during the experiments. Quantitative analysis of the microtome images was performed in the same standard segments used for perfusion quantification according to previously described methods [29]. In brief, the cryomicrotome images with their superior spatial resolution depending on slice thickness (range between hearts 59–100 μm) and pixel density of the digital camera (4000 × 4000 pixel Apogee Alta U-16 digital camera, USA; equipped with a variable 70–180 mm focus lens, Nikon, The Netherlands) effectively provided a point cloud of microspheres. To assess microsphere deposition, this point cloud was aligned with the heart geometry in the CMR images via image registration - a process of anatomical landmark-based rigid registration, followed by manual fine-tuning alignment of anatomical features. Microsphere flow was quantified as a function of segment volume, arterial flow rate, and microsphere count fraction [29]. Accurate rigid registration was achieved by using a combination of anatomical landmark-based rigid registration (i.e., identifying the aortic valve, the LV apex, and the proximal LAD) and fine manual rigid transformation adjustments using the 3D visualisation software CMGUI (OpenCMISS Continuum Mechanics, Imaging, Signal processing and System identification; http://www.cmiss.org/cmgui). This allowed exact alignment of the upper and lower right ventricular (RV) insertion points in both images and thus precise overlay of the CMR and microsphere images (Figure 2). Each AHA segment in the MR perfusion images (with known slice thickness) then defined a volume within which spatially co-registered microspheres were counted.

Flow was calculated in mL/min/g of tissue from the following equation:

$$ \frac{N_s}{M_s}\times \frac{F_t}{N_t} $$

where N_s_ is the number of microspheres counted in a segment, N_t_ is the total number of microspheres injected, F_t_ is the total arterial input flow rate in mL/min, and M_s_ is the mass of a segment in grams. Segment mass was derived from segment volume, which was calculated from a fine 3D binarised left ventricular mesh. To quantify microspheres circumferentially in the same standard segments used for quantitative perfusion CMR analysis, the LV centroid and anterior RV insertion point were identified in each perfusion slice, with circumferential segments then defined every 60 degrees around the centroid allowing very accurate alignment of the respective perfusion and cryomicrotome segments (Figure 2).

### Statistics

Data analysis was performed with IBM SPSS statistics for Mac 20.0.0 (SPSS Inc., Chicago, Illinois, USA). Continuous data are expressed as the mean ± standard deviation (SD). To compare quantitative perfusion measurements of the different algorithms with CMR and microspheres we used linear regression analysis and the method proposed by Bland and Altman [38]. The Fisher z transformation was used to compare the correlation strength of the individual algorithms with the microsphere reference-standard. To correct for multiple comparisons a Bonferroni adjustment for post hoc analysis was performed.

The paired sample t-test was used to assess the mean differences between perfusion measurements based on CMR and with microspheres. Intra-observer variability of perfusion CMR analysis was assessed using the coefficient of variation (CV) defined as the ratio of the SD to the mean of the differences between the measurements. A p-value of <0.05 was considered statistically significant. P-values of less than 0.008 remained significant after Bonferroni correction for multiple comparisons.

## Results

**Figure 3:**
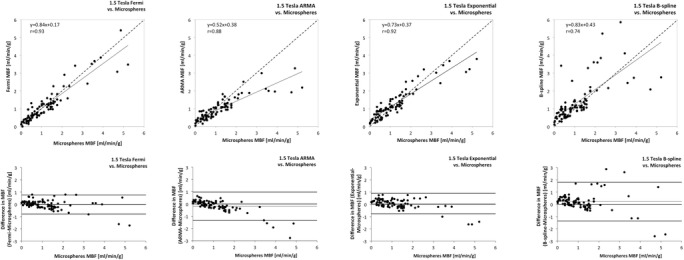
**Agreement of quantitative perfusion analysis using different algorithms with the reference-standard of microsphere derived quantitative perfusion at 1.5 Tesla.** The top row shows the correlation of CMR derived quantitative perfusion analysis using different algorithms with the reference-standard of microsphere derived quantitative perfusion (Microspheres MBF). The bottom row shows the agreement between CMR derived perfusion and Microspheres MBF using the Bland Altman analysis. There was good correlation with minimal error between CMR derived quantitative perfusion analysis and microspheres, which was most evident for Fermi function deconvolution (left hand side).

**Figure 4:**
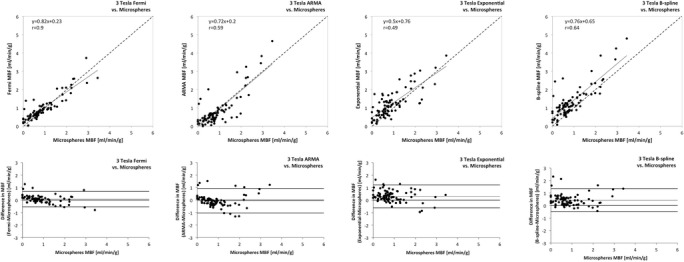
**Agreement of quantitative perfusion analysis using different algorithms with the reference-standard of microsphere derived quantitative perfusion at 3 Tesla.** The top row shows the correlation of quantitative perfusion analysis using different algorithms with the reference-standard of microsphere derived quantitative perfusion (Microspheres MBF). The bottom row shows the agreement between CMR derived perfusion and Microspheres MBF using the Bland Altman analysis. There was excellent correlation with minimal error between CMR derived quantitative perfusion analysis and microspheres using Fermi function deconvolution (left hand side) as opposed to moderate correlation using the other algorithms.

All the explanted hearts recovered electrical function upon reperfusion. In case of reperfusion arrhythmias, defibrillation was performed to achieve sinus rhythm. Hearts were shocked for 3 ± 2 times on average. All hearts remained reasonably stable throughout the perfusion experiments. Heart Rate was 71 ± 15. The heart weight was 272 ± 19 g. The external roller pump blood flow was set to 238 ± 46 mL/min. Further measurements were taken during reduced flow at 118 ± 20 mL/min and during adenosine induced hyperemia with 392 ± 47 mL/min. These flow rates constituted an overall LV perfusion of 0.89 ± 0.18, 0.45 ± 0.1 and 1.47 ± 0.16 mL/min/g LV, respectively. On a segmental level, perfusion estimates derived from the different techniques based on CMR SI-time curves were compared to those obtained with microspheres. Figure 3 shows the relation between segmental microsphere and CMR derived perfusion for 1.5 Tesla and Figure 4 for 3 Tesla.

All CMR derived MBF estimates correlated significantly with those obtained by microspheres (Table 1). The best correlation was achieved with Fermi function deconvolution both at 1.5 Tesla (r = 0.93, p < 0.001) and at 3 Tesla (r = 0.9, p < 0.001, Table 1).

**Table 1:** **The table shows the correlation strength of the individual algorithms with the reference-standard of microspheres**

	**1.5 Tesla**	**3 Tesla**	**p-value**
Algorithm	r	r	
Fermi	0.93	0.9	<0.001
ARMA	0.88	0.59	<0.001
Exponential	0.92	0.49	<0.001
B-spline	0.74	0.64	<0.001

Fermi function deconvolution correlated significantly better with the microspheres as compared to all other methods at 3 Tesla (p < 0.002, Table 2). Whilst it was superior to B-spline at 1.5 Tesla (p = 0.001) there was no difference with exponential deconvolution and ARMA at 1.5 Tesla in terms of correlation strength (p > 0.05, Table 2).

**Table 2:** **Comparison of the correlation coefficients of the individual algorithms**

	**1.5 Tesla**				**3 Tesla**			
Algorithm	Fermi	ARMA	Exponential	B-spline	Fermi	ARMA	Exponential	B-spline
Fermi	p = 1	p = 0.06	p = 0.64	**p = 0.001**	p = 1	**p < 0.001**	**p < 0.001**	**p = 0.001**
ARMA	p = 0.06	p = 1	p = 0.16	p = 0.04	**p < 0.001**	p = 1	p = 0.36	p = 0.6
Exponential	p = 0.64	0.16	p = 1	**p = 0.001**	**p < 0.001**	p = 0.36	p = 1	p = 0.15
B-spline	**p = 0.001**	0.04	**p = 0.001**	p = 1	**p = 0.001**	p = 0.6	p = 0.15	p = 1

The table shows the results of the comparison of the correlation coefficients of the individual algorithms. Fermi function deconvolution had the strongest r-value at both field strengths. In comparison with the other methods it performed significantly better at 3 Tesla. At 1.5 Tesla it performed significantly better than B-spline deconvolution but not than ARMA and exponential deconvolution. The comparison of the correlation strength of the individual algorithms with the microsphere reference-standard was based on Fisher z transformation. P values of less than 0.008 remained significant after Bonferroni correction for multiple comparisons.

The Bland Altman analysis and 95% confidence intervals of the difference showed excellent results for Fermi with minimal overestimation of perfusion at 3 Tesla (mean difference −0.01 ± 0.4 mL/min/g at 1.5 Tesla and 0.07 ± 0.31 mL/min/g at 3 Tesla; see Table 3). ARMA showed minimal underestimation of perfusion at 3 Tesla (mean difference −0.05 ± 0.74 mL/min/g) and moderate underestimation at 1.5 Tesla (mean difference −0.18 ± 0.59 mL/min/g). Exponential deconvolution showed excellent results at 1.5 Tesla (mean difference 0.06 ± 0.43 mL/min/g), however moderate overestimation of perfusion at 3 Tesla (mean difference 0.3 ± 0.74 mL/min/g). B-spline deconvolution showed moderate overestimation of perfusion at both field strengths (mean difference 0.23 ± 0.81 mL/min/g at 1.5 Tesla and 0.43 ± 0.68 mL/min/g at 3 Tesla; Figures 3 and 4, Table 3).

**Table 3:** **Mean perfusion values based on microspheres and CMR for both field strengths**

**Field strength**	**Modality**	**Mean [mL/min/g]**	**Mean difference and CI (95%) of the difference**	**p-value**
1.5 Tesla	Microspheres	1.17 ± 1.05		
	Fermi	1.15 ± 0.96	−0.01 ± 0.4 (−0.1-0.07)	0.74
	ARMA	0.99 ± 0.62	−0.18 ± 0.59 (−0.3-(−0.06))	0.04
	Exponential	1.22 ± 0.83	0.06 ± 0.43 (−0.03-0.14)	0.22
	B-spline	1.39 ± 1.17	0.23 ± 0.81 (0.06-0.39)	0.009
3 Tesla	Microspheres	0.9 ± 0.72		
	Fermi	0.97 ± 0.66	0.07 ± 0.31 (0.002-0.14)	0.43
	ARMA	0.85 ± 0.88	−0.05 ± 0.74 (−0.21-0.1)	0.5
	Exponential	1.21 ± 0.74	0.3 ± 0.74 (0.14-0.46)	<0.001
	B-spline	1.34 ± 0.86	0.43 ± 0.68 (0.29-0.58)	<0.001

Data is presented as mean ± standard deviation (SD) along with p-values indicating statistical significance based on the paired t-test. (CI)- Confidence Intervals; (CMR)-cardiovascular magnetic resonance.

There was a trend towards more pronounced error (i.e. underestimation or overestimation of perfusion) at higher flow values (Table 4). Whilst Fermi showed the least error amongst algorithms at high flow values the error was most pronounced for ARMA deconvolution at 1.5 Tesla.

**Table 4:** **Mean perfusion values based on microspheres and CMR for both field strengths for microsphere perfusion values ≥ 2**

**Field strength**	**Modality**	**Mean [ml/min/g]**	**Mean difference and CI (95%) of the difference**	**p-value**
1.5 Tesla	Microspheres	3.36 ± 1.11		
	Fermi	3.16 ± 1	−0.2 ± 0.85 (−0.74-0.33)	0.42
	ARMA	2.2 ± 0.5	−1.18 ± 1.02 (−1.84-(−0.53))	0.02
	Exponential	2.93 ± 0.61	−0.43 ± 0.81 (−0.94-0.08)	0.91
	B-spline	3.59 ± 1.5	0.23 ± 1.84 (−0.94-1.4)	0.67
3 Tesla	Microspheres	2.57 ± 0.46		
	Fermi	2.51 ± 0.57	−0.07 ± 0.52 (−0.5-0.37)	0.72
	ARMA	3.03 ± 1	0.46 ± 0.91 (−0.31-1.22)	0.2
	Exponential	2.4 ± 0.88	−0.17 ± 0.68 (−0.74-0.4)	0.5
	B-spline	3.34 ± 0.94	0.77 ± 0.78 (0.12-1.42)	0.03

Data is presented as mean ± standard deviation (SD) along with p-values indicating statistical significance based on the paired t-test. (CI)- Confidence Intervals; (CMR)- cardiovascular magnetic resonance.

Intra-observer variability differed between algorithms. Whilst B-spline showed minimal intra-observer variability at both field strengths, the intra-observer variability of Fermi deconvolution was minimal at 1.5 Tesla and moderate to good at 3 Tesla. Exponential deconvolution showed little intra-observer variability at both field strengths whereas ARMA showed some variability at both scanners (Table 5).

**Table 5:** **Intra-observer variability of CMR quantitative perfusion analysis as expressed by the coefficient of variation (CV)**

**Algorithm**	**CV at 1.5 T**	**CV at 3 T**
Fermi	6%	19%
ARMA	27%	32%
Exponential	14%	10%
B-spline	3%	7%

## Discussion

The current study yielded several important findings. Firstly, we demonstrated that Fermi function deconvolution quantitative magnetic resonance perfusion analysis accurately assesses true myocardial blood flow at 1.5 and 3 Tesla using state of the art MR perfusion acquisitions. Secondly, amongst four widely used perfusion quantification algorithms Fermi function reaches the best correlation coefficient and least error at both field strengths.

These findings are based on the utilisation of an explanted heart model in a controlled environment where myocardial blood flow is known and its distribution over time within the myocardium is quantified with CMR and validated versus a microsphere deposition method [29]. The availability of this reference-standard allows the identification of underestimation or overestimation of perfusion by a given CMR quantification algorithm. It is important to note that the microsphere method we have employed arguably provides superior accuracy to previous methods, where hearts needed to be physically dissected into segments corresponding to those used for flow quantification from the perfusion CMR images [39–41]. In contrast to this, the current method provides anatomical alignment of heart geometry from two imaging modalities for the comparison of microspheres with perfusion CMR. The current work shows that CMR derived quantitative perfusion imaging assessed with either Fermi, ARMA, Exponential or B-spline deconvolution correlates with fluorescently-labelled microspheres both at 1.5 and 3 Tesla. There is however a difference in the performance of these algorithms. Whilst Fermi performs best at both field strengths, B-spline shows the biggest error at both field strengths. In the current work Fermi function deconvolution is statistically significantly superior to all other algorithms at 3 Tesla. At 1.5 Tesla, although Fermi function reaches the highest correlation coefficient of all algorithms, it does not reach statistically significant superiority over ARMA and exponential deconvolution. Fermi, ARMA and exponential deconvolution have been shown to be less sensitive to noise as compared to B-spline in an ex-vivo, synthetic perfusion phantom [34]. In this phantom the perfusion is known in the whole myocardial compartment [42] and amongst the aforementioned algorithms Fermi showed the least variability of error under very well controlled experimental conditions [34]. It is interesting to speculate whether the slightly lower perfusion values measured with microspheres or the slightly higher observer variability had an impact on the worse performance of ARMA, Exponential and B-spline deconvolution at 3 Tesla. Notwithstanding these considerations the fact that the performance of all algorithms at both field strengths slightly decreased at higher perfusion values and Fermi showed excellent performance at both field strengths, which is in line with previous investigations showing excellent correlations of Fermi with microspheres both at 1.5 and 3 Tesla [39], would argue against this theory. In line with this evidence the results of the current paper add further weight to the value of Fermi, which has also been shown to correlate with FFR with excellent accuracy for the detection of significant CAD using a similar sequence as employed in the present paper at 3 Tesla [18].

Clinically it is important that quantification algorithms have good performance at both field strengths. Higher spatial resolution MR perfusion acquisitions were shown to offer greater visual diagnostic accuracy at 1.5 Tesla most likely due to the increased detection of more subendocardial ischaemia [43]. The higher spatial resolution present at 3 Tesla compared to 1.5 Tesla should allow even better detection of subendocardial ischaemia and the detection of the severity of the ischaemia in addition to its pure present and extent. Hsu et al. demonstrated that perfusion gradients between subendocardial and subepicardial perfusion can be detected using low spatial resolution techniques and pixel wise quantification strategies in patients [40]. This technique has also been developed for 3 Tesla and interestingly, Fermi function deconvolution also showed the best results in pixel-wise analysis at 3 Tesla [34]. On the other hand, there is recent evidence from our group that standard kt-balanced turbo field echo (kt-BTFE) based perfusion CMR at 1.5 Tesla has a similar diagnostic accuracy for quantitative myocardial perfusion reserve analysis as compared to the clinical reference-standard of quantification PET in patients with CAD [24]. This sequence has also been selected in a major ongoing perfusion CMR clinical trial (MR-INFORM, clinicaltrials.gov NCT01236807) [44]. Our study shows that it may also allow accurate quantitative perfusion analysis. Sub-studies of the MR-INFORM trial aiming to investigate its diagnostic accuracy and prognostic implications will certainly be of interest.

The detected differences in quantitative perfusion assessment using different algorithms are likely to be real because some unknown variables, that occur in patient or in living animal studies, affecting quantitative perfusion assessment do not exist in the explanted pig heart model used in this study. These include the known intracoronary blood flow rates and the fact that the AIF is directly taken from the tubing connecting to the coronaries. Quantification in the explanted heart is less susceptible to external influences such as heart rate, ejection fraction or cardiac output, factors that are all known to heavily influence the shape of the AIF bolus measured in the left ventricular cavity in patients. In fact, in the current model we measure the AIF of the bolus that directly and completely washes into the myocardium. The current model may therefore be useful as a new reference standard for quantitative perfusion validation using CMR especially given the fact that techniques are very easily translatable to patients.

Given the mounting evidence of the use of quantitative perfusion analysis to detect early and subtle changes for various diseases, we believe that Fermi function deconvolution represents an accurate and robust technique that can be recommended at 1.5 and 3 Tesla using current state of the art perfusion sequences. Whether this approach or recently introduced novel CMR based perfusion methodology such as the gradientogram method [45] or pixel wise assessment of perfusion are most accurate will need to be clarified in future studies [34,40].

### Limitations

The fact that the explanted heart is less physiological and free from external influences such as changes in heart rate and cardiac output makes it an ideal validation platform for quantitative perfusion. However, this model may oversimplify in vivo imaging conditions, where the AIF cannot be measured directly and which suffer from artifacts due to breathing motion during stress. Consequently, within in-vivo perfusion quantification experimental settings both CMR and microsphere blood flow quantification techniques still require assumptions (e.g. adequate reference flow measurements with microspheres and adequate AIF measurements with CMR) and therefore neither CMR in the current version nor microspheres may provide absolute fully quantitative measurements. Furthermore, future work should study perfusion under fully working-heart conditions to achieve scenarios that even closer resemble in-vivo physiology to investigate the influence of different filling or working states of the heart on quantitative perfusion analysis.

The current paper describes validation of different quantification algorithms based on AHA segments. Future studies will also need to determine transmural differences of perfusion from subendocardium to subepicardium and to define the diagnostic merits of such assessments at a resolution beyond the AHA segmental classification.

## Conclusions

CMR derived quantitative blood flow estimates accurately assess true myocardial blood flow in a controlled animal model. There are inherent differences between the quantification algorithms used at 1.5 Tesla and 3 Tesla. Fermi function deconvolution qualifies as the most accurate at both field strengths and may represent a technique that allows perfusion CMR quantification to develop into a useful clinical tool.

## Abbreviations

AIF: Arterial input function
ARMA: Autoregressive moving average modelling
BLAST: Broad linear speed up technique
CAD: Coronary artery disease
CFR: Coronary flow reserve
CI: Confidence intervals
CMR: Cardiovascular magnetic resonance
CV: Coefficient of variation
FFR: Fractional flow reserve
LV: Left ventricle
MBF: Myocardial blood flow
MI: Myocardial infarction
MR: Magnetic resonance
PET: Positron emission tomography
SD: Standard deviation
SI: Signal intensity
